# Diagnostic accuracy of cognitive screening tools validated for older adults in Iran: a systematic review and meta-analysis

**DOI:** 10.1186/s12877-024-04963-w

**Published:** 2024-05-14

**Authors:** Leila Kamalzadeh, Gooya Tayyebi, Behnam Shariati, Mohsen Shati, Vahid Saeedi, Seyed Kazem Malakouti

**Affiliations:** 1 Geriatric Mental Health Research Center, Department of Psychiatry, School of Medicine, Iran University of Medical Sciences, Tehran, Iran; 2 Department of Psychiatry, School of Medicine, Mazandaran University of Medical Sciences, Sari, Iran; 3 Mental Health Research Center, Psychosocial Health Research Institute, Department of Psychiatry, School of Medicine, Iran University of Medical Sciences, Tehran, Iran; 4 Mental Health Research Center, Psychosocial Health Research Institute, Iran University of Medical Sciences, Tehran, Iran; 5 School of Public Health, Department of Epidemiology, Iran University of Medical Sciences, Tehran, Iran; 6 Pediatric Endocrinology and Metabolism Department, School of Medicine, Iran University of Medical Sciences, Tehran, Iran; 7 Geriatric Mental Health Research Center, School of Behavioral Sciences and Mental Health, Iran University of Medical Sciences, Tehran, Iran

**Keywords:** Cognitive screening, Diagnostic accuracy, Aged, Risk of Bias, Iran

## Abstract

**Background:**

This systematic review aims to comprehensively assess the diagnostic accuracy of cognitive screening tools validated for older adults in Iran, providing evidence-based recommendations for clinicians and researchers.

**Methods:**

A comprehensive search in March 2023 across Web of Science, PubMed, Scopus, ScienceDirect, SID, IranMedex, and IranDoc, enhanced by hand-searching references and Google Scholar, identified cross-sectional studies on cognitive screening in Iranian seniors. We assessed diagnostic accuracy, cognitive domains, and test strengths and weaknesses. A bivariate random-effects meta-analysis provided summary estimates and 95% confidence intervals, illustrated in forest plots.

**Results:**

Our review, derived from an initial screening of 38 articles, focused on 17 studies involving 14 cognitive screening tools and participant counts from 60 to 350, mostly from specialized clinics. The MMSE was the only tool examined in at least three studies, prompting a meta-analysis revealing its sensitivity at 0.89 and specificity at 0.77 for dementia detection, albeit amidst significant heterogeneity (I^2 > 80%). ACE-III demonstrated the highest diagnostic accuracy for MCI and dementia, while MoCA’s performance was deemed adequate for MCI and excellent for dementia. High bias risk in studies limits interpretation.

**Conclusion:**

This review identifies key cognitive tools for dementia and MCI in Iranian older adults, tailored to educational levels for use in primary and specialized care. It emphasizes the need for further validation to enhance diagnostic precision across diverse settings, within a concise framework prioritizing brevity and accuracy for clinical applicability.

**Supplementary Information:**

The online version contains supplementary material available at 10.1186/s12877-024-04963-w.

## Introduction

The process of dementia can be described as a continuum with a long preclinical phase without clinical symptoms, an early clinical phase in which mild cognitive impairment (MCI) is present, and a dementia phase [1]. Cognitive impairment can significantly impact the quality of life, functional independence, and health outcomes of older adults and their caregivers [2]. The global aging trend has resulted in a rapid increase in the prevalence of dementia, which is expected to affect more than 78 million people worldwide by 2030. Thus, dementia is a major public health challenge, especially in developing countries where two-thirds of cases occur, yet only 10% of research on the disease has been conducted [3].

The early and accurate detection of cognitive impairment is essential for providing effective interventions, such as pharmacological treatments, cognitive rehabilitation, and lifestyle modifications, that can delay or prevent the progression of cognitive decline and improve the well-being of older adults and their families [4, 5]. However, the diagnosis of cognitive impairment can be challenging due to the heterogeneity of its causes, symptoms, and course [6, 7]. Moreover, various factors can influence cognitive impairment, such as age, education, culture, language, and comorbidities [8, 9].

Cognitive screening tools are standardized instruments that assess older adults’ cognitive status in various settings, such as primary care, geriatric clinics, or community-based programs [1]. These tools can also be used to monitor cognitive functioning changes over time and evaluate interventions’ effects [10]. Various assessment scales have been developed to screen for cognitive, behavioral, and functional changes in patients with cognitive decline. However, not all cognitive screening tools are equally valid, reliable, accurate, and useful for different populations and purposes [11]. It is crucial to consider that many of these tools were developed and validated primarily for populations in developed countries, potentially limiting their applicability in developing countries due to cultural and educational disparities [12]. Hence, selecting a cognitive screening tool should carefully consider factors such as accuracy, feasibility, and suitability for the specific population and context of interest.

Iran, as a developing nation, is witnessing a significant demographic shift towards an aging population. The latest census data revealed that individuals aged 60 and over constituted 9.3% of the population in 2016, a figure projected to more than double to 21.7% by 2050 [13]. Research into the incidence and prevalence of cognitive disorders within this demographic is sparse, with existing studies primarily conducted at provincial or district levels. These studies generally indicate a prevalence of cognitive impairment among Iranian seniors ranging from 3.7 to 13%, suggesting a notably high rate of occurrence [14–16].

One of the few studies to assess the national prevalence of dementia in Iran by Sharifi et al. found that 7.9% of the population over 60 years old suffer from dementia, with contributing factors including diabetes, depression, illiteracy, and advancing age. Notably, this study also revealed that a mere 21.2% of those with dementia had been formally diagnosed [17]. Furthermore, a recent systematic review identified a 2.3% prevalence of Alzheimer’s disease in Iranians aged 67 to 78 years, highlighting age, genetic factors, depression, and hypertension as key risk factors [18]. Analysis of 2019 Global Burden of Disease data positions Iran, Turkey, and Bahrain as having the highest age-standardized incidence rates (ASIRs) of AD and other dementias per 100,000 among 204 countries (Iran’s ASIR: 0.11). This highlights a significant public health challenge [19].

Multiple attempts have been made to modify, validate, or develop cognitive screening tools for older adults in Iran. Currently, diverse validated screening tools with distinct methods, reliability, and results are used in outpatient/inpatient clinics and research environments, highlighting the need for a comprehensive assessment of their precision. Hence, this systematic review aims to offer a comprehensive overview of the existing evidence regarding the diagnostic accuracy measures of validated cognitive screening tools for older Iranian adults, aiding clinicians and researchers in selecting the most suitable instrument for their requirements.

## Methods

This systematic review was conducted according to the principles outlined in the Cochrane Handbook for Systematic Reviews of Diagnostic Test Accuracy [20] and the preferred report items of an in-development reporting guideline for systematic reviews of outcome measurement instruments (PRISMA-COSMIN for Outcome Measurement Instruments (OMIs) [21].

### Formulation of the PICO question

To guide this review, we structured our inquiry around the PICO framework:

- P (participants/ population): Iranian seniors aged 60 and older.
- I (index tests/intervention): Validated Persian version of cognitive screening tools for the assessment/screening of cognitive impairment.
- C (comparator/reference tests): The gold standard diagnostic tools, including the Diagnostic and Statistical Manual of Mental Disorders (DSM III to DSM-5-TR) [22] and the International Classification of Diseases (ICD-8 to ICD-11) for diagnosis of dementia/major neurocognitive disorder and MCI/minor neurocognitive disorder [23].
- (outcome): Measures of diagnostic accuracy (sensitivity, specificity, positive/negative predictive values, likelihood ratios, and diagnostic odds ratios) and cognitive domain assessment.

### Eligibility criteria

Inclusion criteria:

- Cross-sectional studies examining the psychometric/diagnostic properties of at least one instrument (index test) for the assessment/screening of cognitive impairment (target condition) in Iranian seniors (adults aged 60 and older).
- Studies that used a gold standard diagnostic tool such as the Diagnostic and Statistical Manual of Mental Disorders (DSM III to DSM-5-TR) [22] or the International Classification of Diseases (ICD-8 to ICD-11) [23] criteria to confirm cognitive impairment were prioritized.

Exclusion criteria:

- Studies focusing on participants selected based on a specific disease or medical field, such as heart failure or Parkinson’s disease.
- Studies utilizing measures of daily living activities, functional status, self-administered tests, caregiver/informant-rated tests, and telephone-based or computerized/web-based tests.
- Studies that only assess subtests of cognitive screening instruments, have unclear validity information, or provide insufficient data.

### Information sources and search strategy

In March 2023, a comprehensive electronic literature search was conducted utilizing seven databases, including Web of Science, PubMed, Scopus, ScienceDirect, the Scientific Information Database (SID), IranMedex, and the Iranian Research Institute for Information Science and Technology (IranDoc). The search was supplemented by hand-searching references of retrieved papers and searching Google Scholar.

The search entailed articles in both English and Persian published up until the time of the search that contained a combination of keywords and MeSH terms such as ((((“older adults” OR “elderly” OR “senior” OR “geriatric”) AND (“Iran” OR “Iranian” OR “Farsi” OR “Persian”)) AND ((“cognitive screening tools” OR “neuropsychological assessments” OR “cognitive tests”))) AND ((“cognitive disorders” OR “cognitive impairment” OR “dementia” OR “MCI” OR “neurocognitive disorders”))) AND ((“diagnostic accuracy” OR “sensitivity” OR “specificity” OR “PPV” OR “NPV” OR “psychometric properties” OR “validation”)). The search strategies for the databases are included in Appendix 1.

All searches were imported into the Mendeley reference management system, and duplicates were removed.

### Selection process

The titles and abstracts of all identified articles were screened for eligibility by two independent reviewers (G.T. and B.S.) who then evaluated the full-text articles for inclusion. In the event of disagreements, a third reviewer (M.S. or V.S) was consulted to facilitate resolution.

### Data collection process and data items

Data regarding essential measures of diagnostic accuracy, including sensitivity, specificity, positive and negative predictive values (PPV/NPV), and positive and negative likelihood ratios (LR+/LR-), for discriminating between normal cognitive function, MCI, and dementia/major neurocognitive disorder were extracted or calculated where possible by two independent reviewers. In cases where studies reported PPVs and NPVs, we also calculated the Number Needed for Screening Utility (NNSU) as the reciprocal of the Summary Utility Index (SUI), which is derived from the sum of the Clinical Utility Index for ruling in (CUI + = sensitivity x PPV) and ruling out (CUI- = specificity x NPV) a diagnosis. This calculation aims to gauge the test’s efficiency in confirming or excluding a diagnosis. NNSU values span from infinity (indicating no screening utility) to 0.5 (signifying perfect screening utility), with lower values preferred. NNSU is further classified qualitatively into excellent (0.5 ≤ NNSU ≤ 0.62), good (0.62 < NNSU ≤ 0.78), adequate (0.78 < NNSU ≤ 1.02), and poor (> 1.02) to provide a comprehensive assessment of a screening test’s clinical utility [24].

Additionally, the cognitive domains assessed by each test and their respective advantages and disadvantages were identified. When data were missing, unclear, or incompletely reported in a study, efforts were made to contact the authors to obtain the necessary information.

It is important to note that the positive and negative predictive values of a diagnostic test are influenced by the prevalence of the disorder within the study population. Therefore, in the present review, likelihood and diagnostic odds ratios were also calculated.

### Study risk of bias assessment

Two reviewers (G.T. and B.S.) assessed, discussed, and reached a consensus on the methodological quality of each included study using the recommended quality assessment tool for Diagnostic Accuracy Studies (QUADAS-2). The QUADAS-2, an updated version of the original QUADAS tool, is specifically designed for assessing the risk of bias and applicability in diagnostic accuracy studies. It evaluates four main domains: patient selection, index test, reference standard, and study flow and timing. Risk of bias is examined across all domains, while the first three are also checked for applicability concerns, offering a structured method for the critical appraisal of studies to ensure systematic and reliable assessment of evidence quality [25].

### Statistical analysis and synthesis of results

The statistical analyses were conducted using Stata statistical software (version 17, Stata-Corp). For each test that was validated by a minimum of three studies, a bivariate random-effects meta-analysis of diagnostic accuracy data was conducted using the “Midas” command in Stata. The summary estimates of sensitivity, specificity, LR+, LR-, and DOR with 95% confidence intervals (CIs) were obtained. Forest plots of the sensitivity and specificity estimates, and a summary receiver operating characteristic (SROC) plot were constructed. An investigation of heterogeneity was performed.

### Registration and protocol

A review protocol with predefined criteria was registered in the PROSPERO international prospective register of systematic reviews (https://www.crd.york.ac.uk/prospero/, registration number CRD42021291784).

## Results

### Study selection and characteristics

**Fig. 1:**
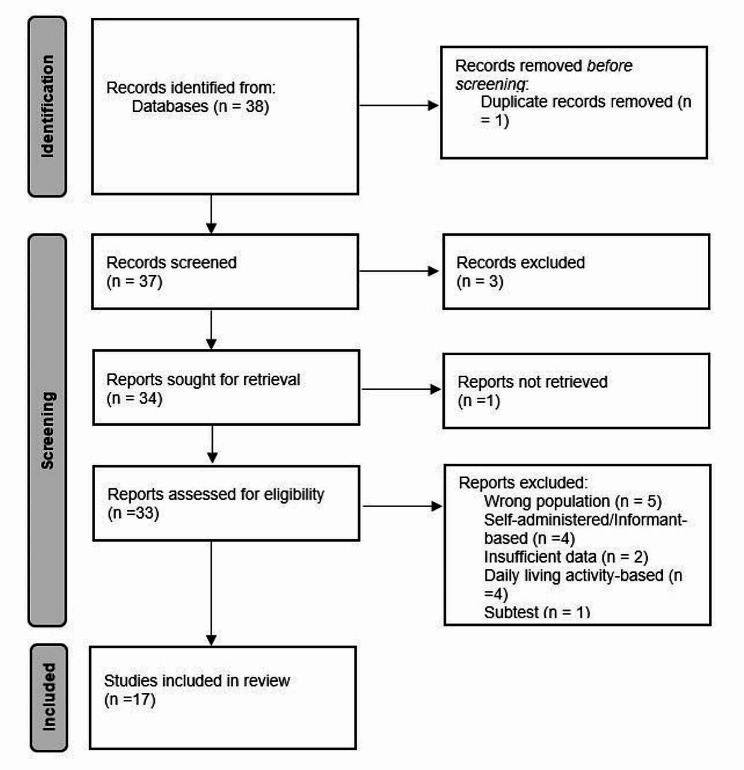
Flow diagram showing the selection of studies for the systematic review and meta-analysis

The systematic search yielded 38 articles, of which 21 were excluded based on predefined criteria. The remaining 17 studies, investigating 14 different instruments, were included in the review. In cases where the same test had been validated among different population groups in Iran, each validation was reported separately to provide a comprehensive analysis. For one study, however, access to the full-text article was unavailable; therefore, information was extracted from the abstract alone. A flow diagram (Fig. 1) was generated to visualize the study selection process, providing a clear overview of the screening and inclusion/exclusion process.

### Risk of bias in studies

**Fig. 2:**
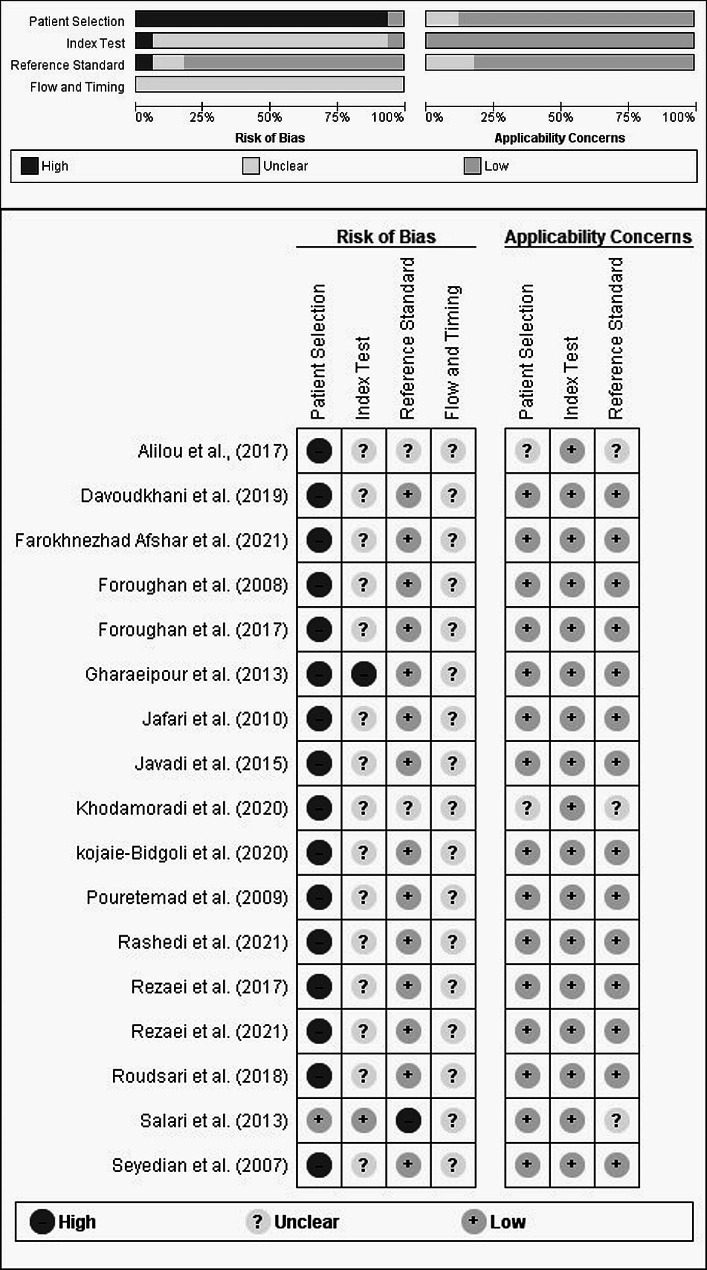
Risk of bias and applicability concerns graph: review authors’ judgments about each domain presented as percentages across included studies

Using QUADAS-2 to evaluate the quality of the four domains, it was observed that most of the studies, except for one (the study by Salari et al. [26], employed a case‒control design. This design choice introduced a significant risk of bias in the patient selection and index test domains. Additionally, a considerable number of studies gathered samples from specialized clinics or nursing centers, potentially limiting the generalizability of their findings to the broader patient population. Two studies lacked a reference standard (the studies by Salari et al. [26] and Khodamoradi et al. [27]), and another had an unclear standard (the study by Aliloo et al. [28]), causing high and unclear bias risks for reference test interpretation. None of the studies reported the time interval or any interventions between the index test and the reference standard, making this aspect ambiguous. Most of the studies had a low concern of applicability to the review question in terms of patient characteristics, index test conduct and interpretation, or reference standard. Three studies (Salari et al. [26]Khodamoradi et al. [27], and Aliloo et al. [28]) had unclear concern of applicability to the review question regarding the patient selection and target condition due to insufficient reporting of reference standard details. The risk of bias and applicability concerns for the seventeen included studies are summarized in Fig. 2.

### Results of syntheses

The included studies varied in size, with participant numbers ranging from 60 to 350 individuals. Most of the studies included patients with cognitive disorders recruited from specialized clinics and healthy controls recruited from their companions and relatives. Two studies sourced their patients from nursing and daycare centers, and one study partly enrolled their sample from patients admitted to the neurology ward. Further characteristics of the included studies and diagnostic test accuracy results for cognitive tests are summarized in Table 1.

**Table 1:** Characteristics of included studies and diagnostic test accuracy results for cognitive tests

Study	Setting	Target condition	Total number of participants	Formal education (years)	Index test	Cutoff	Sensitivity (%)	Specificity (%)	PPV (%)	NPV (%)	LR+	LR-	DOR	Reference standard
Seyedian et al. (2007)	Memory clinic (IDAA)	Dementia	220	≥ 1	MMSE	≤ 22	90	93.5	-	-	13.84	0.10	138.4	DSM-IV
Foroughan et al. (2008)	Memory clinic (IDAA)	Dementia	205	≥ 4	MMSE	≤ 21	90	84	-	-	5.62	0.11	51.09	DSM-IV-TR
Pouretemad et al. (2009)	Memory clinic (IDAA)	MCI and dementia	139	≥ 4	ACE-III	For MCI: 84	93	91	-	-	10.33	0.07	147.57	NINCDS- ADRDA
						For dementia: 78	73	93	-	-	10.42	0.2	52.1	
Jafari et al. (2010)	Memory clinic (IDAA)	Dementia	350	≥ 1	RAVLT	2.5–6.5	89	81	-	-	4.68	0.13	36	DSM-IV
Salari et al. (2013)	Geriatric Nursing home and day-care center	dementia	500	≥ 0	RUDAS	≤ 20	86	79	-	-	4.09	0.17	24.05	None
Gharaeipour et al. (2013)	Neurology ward and outpatient neurology clinic	Dementia	203	≥ 1	3MS	≤ 78	98	81	-	-	8.9	0.02	445	DSM-IV
Javadi et al. (2015)	Memory clinic	Dementia	101	≥ 0	PEACE	≤ 67.5	75.8	97.4	-	-	29.15	0.24	121.45	DSM-IV, NINCS-ADRDA
Foroughan et al., (2017)	Memory clinic (IDAA)	Dementia	202	≥ 1	AMTS	≤ 6	99	85	-	-	6.60	0.01	660	DSM-IV-TR
Rezaei et al. (2017)	Neurology clinics and day care centers	Dementia	100	≥ 1	Mini-Cog	≤ 2	88	62.8	-	-	2.31	0.19	12.15	DSM-5
Aliloo et al., (2017)	Geriatric Nursing Home	Dementia	60	Unclear	ACE-III	≤ 75	99	95	-	-	19.8	0.01	1980	Unclear
Roudsari et al. (2018)	Memory clinic (IDAA)	Dementia	74	≥ 4	CDT	≤ 3	90	73	69	91	3.33	0.13	25.61	DSM-IV-TR
Davoudkhani et al. (2019)	Memory clinic	Dementia	119	≥ 0	PIMIS	≤ 5	60	91	0.63	0.90	6.66	0.43	15.48	DSM-IV-IR, NINCDS-ADRDA-AD
Khodamoradi et al.(2020)	Unclear	Dementia	310	0	MMSE	≤ 21.5	95	72	-	-	1.33	0.06	22.16	Unclear
kojaie-Bidgoli et al. (2020)	Neurology and geriatric clinics	Dementia	156	≥ 0	SPMSQ	Illiterate ≥ 4	86.4	88.2	93.6	100	7.32	0.15	48.8	DSM-5
						Literate ≥ 3	83	93.7	80.8	93.6	13.17	0.18	73.16	
Farokhnezhad Afshar et al. (2021)	Memory clinic	MCI and dementia	114	≥ 0	AQT	For MCI: Color 43.5 s, Form 52 s, and Color-Form 89 s	95–98	11–38	-	-	1.06–1.58	0.45 − 0.05	2.35–31.6	DSM-5
						For Dementia: Color 62.50 s, Form 111 s, Color-Form 197.50 s	87–96	22–59	-	-	1.11–2.34	0.59 − 0.06	1.88-39	
Rashedi et al. (2021)	Memory clinic	MCI and dementia	120	≥ 1	MoCA	For MCI: 22	86.4	70	81	77.8	2.88	0.19	15.15	DSM-5
						For dementia: 20	94.9	90.5	96.6	94.9	9.98	0.05	199.6	
					MMSE	For MCI: 26	72.9	67.5	76.8	62.8	2.24	0.40	5.6	
						For dementia: 21	61.9	38.1	81.9	100	1	1	1	
Rezaei et al. (2021)	Geriatric outpatient clinic	MCI and dementia	92	≥ 0	QMCI	For MCI: <53	79	80	-	-	3.95	0.26	15.19	DSM-5
						For dementia: <38	88	90	-	-	8.8	0.13	67.69	

ACE−III: Addenbrooke’s Cognitive Examination– III; AMTS: Abbreviated Mental Test Score; AQT: A Quick Test of Cognitive Speed; CDT: Clock Drawing Test; DSM: Diagnostic and Statistical Manual of Mental Disorders; IDAA: Iran Dementia and Alzheimer’s Association; MCI: Mild Cognitive Disorder; MMSE: Mini−Mental State Examination; MoCA: Montreal Cognitive Assessment; 3MS: Modified Mini−Mental State Examination; NINCS−ADRDA: National Institute of Neurological and Communicative Disorders and Stroke/Alzheimer’s Disease and Related Disorders Association; PEACE: Persian test of Elderly for Assessment of Cognition and Executive; PIMIS: Persian Version of Illustrated Memory Impairment Screen; QMCI: Quick Mild Cognitive Impairment screen; RAVLT: Rey Auditory−Verbal Learning Test; RUDAS: Rowland Universal Dementia Assessment Scale; SPMSQ: Short Portable Mental Status Questionnaire

### NNSU for identifying mci and dementia

Only Rashedi et al. [29]reported PPVs and NPVs for the MoCA and MMSE scales, allowing for the calculation of NNSU. MoCA demonstrated an adequate NNSU for detecting MCI (0.81) and excellent performance for dementia (0.56). Conversely, MMSE displayed poor NNSU for both MCI (1.03) and dementia (1.13).

Table 2 summarizes the characteristics of each scale, including the cognitive domains covered, advantages, and disadvantages.

**Table 2:** Scale characteristics, cognitive domains covered, advantages, and disadvantages

Cognitive screening test	Administration time(minutes)	Cognitive domains						Advantages	Disadvantages
		Orientation	Attention/Concentration	Memory	Executive functions	Visuospatial abilities	Language		
CDT	≤ 5		✓		✓	✓		Quick, simple, nonverbal, Language and culturally unbiased, not dependent on verbal abilities	Lacks episodic memory testing. Reliance on visuospatial and motor abilities. Affected by the level of education. Limited accuracy in detecting MCI.
Mini-Cog	≤ 5		✓	✓	✓	✓	✓	Quick, simple, Language and culturally unbiased	Reliance on visuospatial and motor abilities. Affected by the level of education. Limited accuracy in detecting MCI
SPMSQ	≤ 5	✓	✓	✓				Quick, simple, less affected by the level of education	Lacks episodic memory testing. Evaluates a limited number of cognitive domains. Limited accuracy in detecting MCI.
Qmci	≤ 5	✓	✓	✓	✓	✓		Quick, simple, broad cognitive domains covered, high accuracy for detecting MCI and dementia	Affected by the level of education
RUDAS	5–10	✓	✓	✓	✓	✓	✓	Simple, less affected by culture, language, and level of education, Comprehensive cognitive domains covered	Limited accuracy in detecting MCI
AQT	5–10		✓		✓			Simple, less affected by culture and language	Limited number of cognitive domains covered. Affected by the level of education (under grade 8). Reliance on motor skills. Limited accuracy in detecting MCI
PMIS	5–10		✓	✓				Simple, less affected by the level of education	Limited number of cognitive domains covered. Limited accuracy in detecting MCI.
AMTS	5–10	✓	✓	✓				Simple, not affected by motor skills	Limited number of cognitive domains covered. affected by the level of education, language, and culture. Limited accuracy in detecting MCI.
MMSE	10–15	✓	✓	✓		✓	✓	Simple, broad range of cognitive domains covered	Affected by the level of education. reliance on motor skills. Limited assessment of executive function. Limited accuracy in detecting MCI.
3MS	10–15	✓	✓	✓	✓	✓	✓	Simple, comprehensive cognitive domains covered	Affected by the level of education. reliance on motor skills. Limited accuracy in detecting MCI.
MoCA	15–20	✓	✓	✓	✓	✓	✓	Comprehensive cognitive domains covered	Affected by the level of education, culture, and language. Reliance on motor skills. Moderate accuracy in detecting MCI.
ACE-III	15–20	✓	✓	✓	✓	✓	✓	Comprehensive cognitive domains covered	Affected by the level of education, culture, and language. Reliance on motor skills
PEACE	15–20	✓	✓	✓	✓		✓	Broad cognitive domains covered, culturally adapted, less affected by the level of education	Limited accuracy in detecting MCI.
RAVLT	20–30		✓	✓				Multiple memory processes assessed	Limited cognitive domains covered. Affected by the level of education, culture, and language. Limited accuracy in detecting MCI.

ACE−III: Addenbrooke’s Cognitive Examination– III; AMTS: Abbreviated Mental Test Score; AQT: A Quick Test of Cognitive Speed; CDT: Clock Drawing Test; MMSE: Mini−Mental State Examination; MoCA: Montreal Cognitive Assessment; 3MS: Modified Mini−Mental State Examination; PEACE: Persian test of Elderly for Assessment of Cognition and Executive; PIMIS: Persian Version of Illustrated Memory Impairment Screen; QMCI: Quick Mild Cognitive Impairment screen; RAVLT: Rey Auditory−Verbal Learning Test; RUDAS: Rowland Universal Dementia Assessment Scale; SPMSQ: Short Portable Mental Status Questionnaire

### Pooled estimation of diagnostic accuracy of the MMSE test

**Fig. 3:**
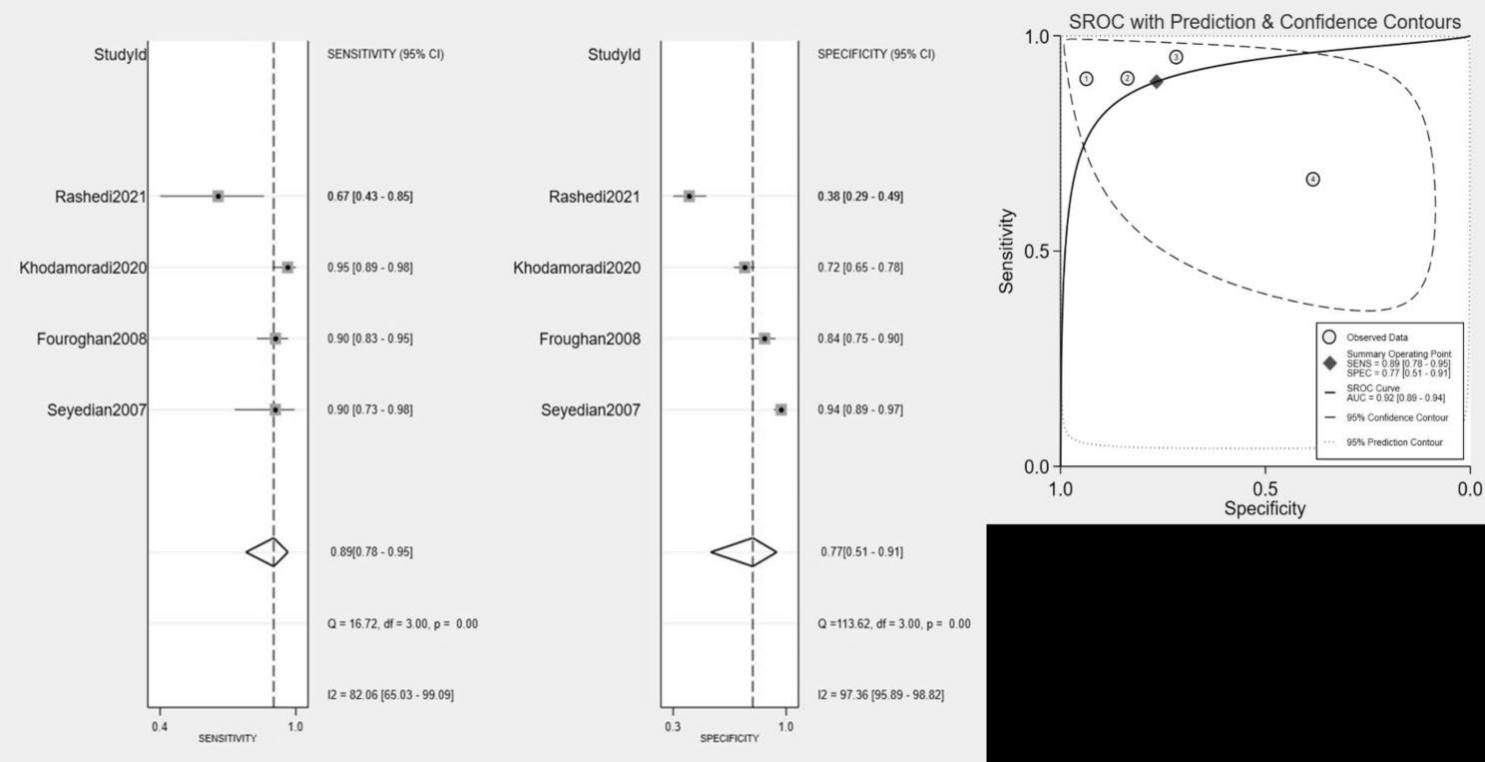
MMSE sensitivity, specificity, and summary receiver operating characteristic for cognitive disorder identification in four studies

To provide context for our focused analysis, it is important to note that among the cognitive screening tools identified, only the MMSE was validated in three or more studies that met our criteria for inclusion in a meta-analysis. The bivariate effect model was utilized to derive pooled values for the MMSE with cutoffs of 21–22, specifically for dementia detection. These values were obtained from the psychometric evaluation conducted in four studies. The pooled sensitivity was 0.89 (95% CI 0.78–0.95), while the pooled specificity was 0.77 (95% CI 0.51–0.91). Additionally, the diagnostic odds ratio (DOR) was calculated as 27 (95% CI 5-166), the positive likelihood ratio (LR+) was 3.8 (95% CI 1.5–9.6), and the negative likelihood ratio (LR-) was 0.14 (95% CI 0.05–0.36). To visually represent the data from each study, forest plots and a summary receiver operating characteristic (SROC) plot were generated (Fig. 3). Substantial heterogeneity was observed in the pooled sensitivity (I^2^ = 82.06, 95% CI 65.3-99.09) and specificity (I^2^ = 97.36, 95% CI 95.89–98.82) (Fig. 3). The AUC, as an overall measure for test performance, was 0.92 (95% CI: 0.89–0.94), indicating the high diagnostic accuracy of this test.

## Discussion

This systematic review was conducted to identify the cognitive tests that have been validated in older Iranian adults and to evaluate the evidence for their accuracy. The initial section of the discussion provides a brief description of the diagnostic accuracy measures of the investigated instruments, categorizing them based on their administration time. Subsequently, the discussion delves into the applicability of these instruments in identifying dementia and MCI in different clinical settings.

### Instruments with shorter assessment time (< 10 min)

#### Clock drawing test (CDT)

The CDT has recently gained popularity as a straightforward, efficient, and acceptable tool for screening cognitive disorders. It assesses a range of cognitive domains that may be impaired early in neurocognitive disorders, such as executive functioning and visuo-constructive skills [30]. It is especially useful in patients with marked verbal impairment or aphasia because it does not depend much on verbal abilities. It is also minimally influenced by language and cultural background [31]. However, the CDT does not assess episodic memory; therefore, it is often combined with other tests [32]. Additionally, research findings have indicated that individuals with no literacy skills demonstrate notably poorer performance on this assessment compared to those who are literate [33]. Among Persian-speaking Iranian elderly individuals, the CDT utilizing Shulman’s six-point scoring method demonstrated a sensitivity of 90% and specificity of 73% when employing a cutoff point of ≤ 3 for detecting dementia. The PPV was 69%, while the NPV was 91% [34].. The calculated LR + was 3.33, the LR- was 0.13, and the DOR was 25.61.

#### Mini-cog

The Mini-Cog has emerged as a valuable brief screening tool for detecting cognitive impairments in primary care settings. This assessment integrates a 3-item recall component with an evaluation of a clock drawing task, encompassing a wide array of cognitive functions such as memory, attention, and executive function. The recall test is scored on a scale of 0 to 3, while the clock drawing task is assigned a score of either 0 or 2. These individual scores are then aggregated to generate a total score that ranges from 0 to 5 [35]. Various studies have reported differing sensitivity levels (76–99%) and specificity (89–93%) for the Mini-Cog in detecting dementia. However, its accuracy in detecting cases of MCI is not as reliable [36].. Furthermore, it should be noted that the Mini-Cog may have limited utility in individuals with low levels of education or illiteracy. A recent study conducted in Iran, focusing on older adults admitted to hospitals, discovered that a significant proportion of Persian-speaking seniors with limited education and normal cognitive functioning faced difficulties in completing the clock drawing task of the Mini-Cog test [37].

The optimal cutoff point for the Persian version of the Mini-Cog for detecting dementia was determined to be 2, with a sensitivity of 88% and a relatively modest specificity of 62.8% [38]. The LR + was calculated as 2.31, the negative LR- was 0.19, and the DOR was 12.15.

#### The short portable mental status questionnaire (SPMSQ)

The SPMSQ evaluates orientation, remote memory, mental control, and attention. However, it does not include a specific assessment of short-term memory, nor does it encompass items related to right hemisphere, occipital, or frontal lobe impairments. The score ranges from 0 to 10 based on the number of incorrect answers [39]. The Persian version validated a cutoff point of 4 for illiterate elderly patients (sensitivity 86.4%, specificity 88.2%, LR + 7.32, LR- 0.15, DOR 48.8) and 3 for literate patients (sensitivity 83%, specificity 93.7%, LR + 13.17, and LR- 0.18, DOR 73.16), showing acceptable accuracy for detecting cognitive impairment in both the illiterate and literate groups. However, the SPMSQ is more accurate in identifying moderate and severe dementia than MCI [40].

#### A quick test of cognitive speed (AQT)

The AQT is a cognitive assessment tool designed to evaluate visual-verbal processing speed. Originally developed for primary care settings, it measures the speed of perception and overall cognitive processing. It can be applied to various languages and cultures and individuals with low education levels [41]. The AQT has demonstrated high sensitivity (87–98%) but low specificity (11–59%) for detecting dementia (DOR 1.88-39) and MCI (DOR 2.35–31.6) in Iranian seniors [42].

#### The quick mild cognitive impairment (Qmci) screen

The Qmci screen is a reliable and brief tool specifically designed to distinguish between individuals with MCI and normal controls. It assesses cognitive functioning across six subtests, covering orientation, working memory, semantic memory, visuospatial ability, and episodic memory [43]. The Persian version of the Qmci demonstrates fair accuracy in identifying MCI and mild dementia, with an area under the curve (AUC) of 0.87. At an optimal cutoff score of < 53/100, it exhibits a sensitivity of 79%, specificity of 80%, LR + of 3.95, LR- of 0.26, and DOR of 15.19). This test accurately identifies moderate to severe dementia, with a sensitivity of 88%, specificity of 90%, LR + of 8.8, LR- of 0.13, and DOR of 67.69 at an optimal cutoff of < 38/100 [44].

#### Rowland universal dementia assessment (RUDAS)

The RUDAS is a cognitive assessment tool suitable for multicultural settings and individuals with limited literacy. It evaluates body part recognition, visuospatial function, reasoning, and memory [45].In the Persian version of the RUDAS, a cutoff score of 20 demonstrates a sensitivity of 86%, specificity of 79%, LR + of 8.9, LR- of 0.02, and DOR of 24.05 for detecting dementia [26].

#### Picture-based memory impairment screen (PMIS)

The PMIS is a concise screening tool that involves four pictures from distinct categories to evaluate delayed free and cued recall. Each picture freely recalled by the individual receives two points, while pictures recalled with cues are awarded one point, resulting in a score range of 0 to 8. Notably, the PMIS does not necessitate the ability to write, exhibits minimal susceptibility to educational and literacy levels, and can be easily administered by trained non-specialists. However, it does not encompass an assessment of executive function and demonstrates limited sensitivity in detecting early-stage dementia and MCI [46]. The PMIS has undergone validation in older Iranian adults, exhibiting a sensitivity of 60%, specificity of 91%, PPV of 63%, and NPV of 90% for detecting dementia. These metrics were established using a cutoff score of 5 [47]. An LR + of 6.66 and an LR- of 0.43 were calculated for this study.

#### Abbreviated mental test score (AMTS)

The AMTS was originally developed and validated in 1972 as a preliminary screening tool designed to identify cognitive impairment in elderly patients. This concise assessment consists of ten items that evaluate intact short- and long-term memory, attention, and orientation abilities [48]. Notably, the AMTS is freely accessible and can be administered quickly and easily, making it suitable for individuals with limited literacy skills. Furthermore, it does not necessitate the use of writing utensils or paper, rendering it appropriate for individuals with visual or physical impairments [48, 49]. However, one of its limitations lies in the requirement for two individuals to be present at the bedside during the assessment for the recognition question. Depending on the chosen cutoff score, the sensitivity and specificity of the AMTS in detecting cognitive impairment have been reported to range from 81 to 96% and 75–86%, respectively [50, 51]. Nevertheless, evidence indicates that the AMTS exhibits a ceiling effect and is less sensitive to milder cognitive deficits [52]. The validation study of the Persian version of the AMTS among older Iranians revealed a total Cronbach’s α coefficient of 0.90. A score of 6 or lower indicates dementia with a sensitivity of 99% and specificity of 85%. The corresponding LR + was 6.60, and the LR- was 0.011, indicating relatively strong diagnostic evidence. In this Persian adaptation, AMTS scores exhibited positive correlations with educational level and male sex, while displaying a negative correlation with age [53].

### Instruments with moderate assessment time (10–15 min)

#### Mini–mental state examination (MMSE)

The MMSE is the most frequently used screening tool for providing an overall measure of cognitive impairment in community, research, and clinical practice. This scale assesses several cognitive domains, with scores ranging from 0 to 30, with higher scores indicating better cognitive function [54]. The MMSE has shown low sensitivity for MCI, does not perform well in assessing executive functions and has limiting floor and ceiling effects [55]. Four studies have investigated the psychometric features of the Persian version of the MMSE in Iranian older adults [27, 29, 56, 57]. The pooled sensitivity, specificity, DOR, LR+, and LR- for the optimal cutoff scores of 21 to 22 for diagnosing dementia were 0.97, 0.87, 242, 7.69, and 0.03, respectively, indicating a high accuracy; however, the accuracy of the MMSE for detecting MCI in Iranian seniors was low, with an LR + of 2.24 and an LR- of 0.4. Age and education levels had significant correlations with MMSE scores in most of the studies reviewed.

#### Modified mini-mental state examination (3MS)

The 3MS is an enhanced version of the MMSE that adds four tasks on long-term memory, abstract thinking, category fluency, and delayed recall. It has a broader score range of 0–100 and maintains the brevity, ease of administration, and objective scoring of the MMSE. However, it is also affected by culture, language, age, physical disability, and education levels [58]. The Persian version of the 3MS had an optimal cutoff score of 78 for detecting dementia with 98% sensitivity and 81% specificity [59]. The LRs were 8.9 for positive test results (LR+) and 0.02 for negative test results (LR-), suggesting a higher accuracy for diagnosing dementia than the MMSE.

### Instruments with longer assessment time (> 15 Min)

#### Montreal cognitive assessment (MoCA)

The MoCA is a 30-point cognitive screening tool designed to address the shortcomings of the MMSE in detecting MCI and mild dementia. It evaluates diverse cognitive domains, including attention, executive function/visuospatial ability, conceptual thinking, free recall, language, and orientation [60]. Healthcare professionals specializing in cognitive assessment are recommended to interpret the results of the MoCA due to its complexity and sensitivity to cognitive impairments [61]. Rashedi et al. established a cutoff score of 22 for the Persian version of the MoCA for identifying MCI, which demonstrated a sensitivity of 86.4%, specificity of 70%, PPV of 81%, NPV of 77.8%, LR + of 2.88, and LR- of 0.19, indicating the MoCA’s satisfactory diagnostic accuracy for detecting MCI. Additionally, the study introduced a cutoff score of 20 for identifying dementia, which exhibited a remarkable sensitivity of 99%, specificity of 94.9%, PPV of 96.6%, NPV of 94.9%, LR + of 9.98, and LR- of 0.05, showing convincing diagnostic accuracy [29].

#### Addenbrooke’s cognitive examination (ACE)-III

The ACE—III is an extended cognitive screening scale developed to overcome the shortcomings of the MMSE with additional items that assess executive functioning, memory, and language in greater depth. It has demonstrated the ability to differentiate individuals with MCI, allows for tracking the progression of cognitive deficits, and shows some utility in distinguishing Alzheimer’s disease from frontotemporal dementia [62]. Nevertheless, ACE-III scores are influenced by age, level of education, and intelligence [63]. The total score of ACE-III is based on a maximum of 100, with higher scores indicating better cognitive functioning [62]. The Persian version of ACE-III had a high accuracy for diagnosing MCI at a cutoff score of 84 (sensitivity: 93%, specificity: 91%, LR + 10.33, LR- 0.07) and dementia at a cutoff score of 78 (sensitivity: 100%, specificity: 96%, LR + 10.42, LR- 0.2) [64]. Additionally, another study reported a cutoff score of 75 for detecting dementia (sensitivity 99%, specificity 95%, LR + 19.8, and LR- 0.01), providing convincing diagnostic accuracy [28].

#### Persian test of elderly for assessment of cognition and executive function (PEACE)

The PEACE is a culturally adapted cognitive screening test proposed to assess the cognitive efficiency of both illiterate and literate older Iranian adults. It consists of 14 items, each of which represents a specific cognitive function, with a maximum score of 91. The 14 items are orientation, praxis, attention and concentration, calculation, memory, similarity, abstract thinking, general information, language, judgment, gnosis, planning (sequencing), problem-solving, and animal naming. A cutoff score of 67.5 was chosen for the optimal diagnosis of dementia by PEACE (sensitivity: 75.8%, specificity: 97.4%, LR + 29.1, LR- 0.24). However, adequate diagnostic accuracy to detect MCI was not shown by the test [65].

#### Rey auditory verbal learning test (RAVLT)

The RAVLT is a five-trial verbal learning and memory test with a delayed recognition component. It assesses the ability to encode, consolidate, store, and retrieve verbal information. While the test is sensitive to verbal learning and recall, it is influenced by age, education, and intelligence. Additionally, given its focus solely on attention/concentration and memory, this test is typically considered a second-tier, domain-specific cognitive assessment, frequently incorporated into comprehensive neuropsychological test batteries [66]. The RAVLT was validated in Iranian older adults, showing convergent validity with the logical subtest of the Wechsler Memory Scale. It had 89% sensitivity and 81% specificity for detecting dementia [67]. The calculated LR + was 4.68, indicating its utility in identifying dementia, while the LR- was 1.08, suggesting a moderate effect on ruling out the presence of dementia.

### Detection of cognitive impairment in primary care and community settings

In primary care and community settings (for example, community-based epidemiological studies), a concise tool must be used to assess cognitive function in older adults and identify those who may require further evaluation. The critical requirements for such tools are ease of use, minimal training requirements, and quick administration [61]. In light of the study’s findings, we propose dividing the elderly population into two groups based on their educational level and recommend suitable cognitive screening tools for each group.

#### Older adults with more than six years of education

For older adults with education beyond the elementary level, we recommend the utilization of the Qmci and RUDAS as primary care screening tools. These instruments effectively assess a broad range of cognitive domains, demonstrate acceptable diagnostic accuracy (with DORs of 67.69 and 24.05, respectively), and can be administered within a brief duration of ten minutes or less. Notably, several systematic reviews have provided robust evidence supporting the diagnostic performance of Qmci and RUDAS in detecting cognitive impairment within primary care settings [68, 69].

#### Illiterate and low-educated older adults

For older adults with limited or no literacy, we recommend using the AMTS, SPMSQ, and PMIS in descending order of diagnostic accuracy. All three tests can be administered swiftly, within ten minutes or less. Among these scales, the AMTS demonstrates remarkable proficiency in identifying cases of dementia (with a DOR of 660). However, the SPMSQ does not encompass assessment of episodic short-term memory, which is typically the initial cognitive domain affected in amnestic mild cognitive impairment and Alzheimer’s disease. It has been evaluated among illiterate Iranian older adults and has shown acceptable diagnostic accuracy (with DORs of 48.8 and 73.16 for illiterate and literate individuals, respectively) [40]. The PMIS evaluates a narrower range of cognitive domains and has demonstrated slightly lower diagnostic accuracy (with a DOR of 15.48) than the abovementioned tests.

Within the context of primary care, it is imperative to recognize that the MMSE stands out as the most extensively researched scale for dementia detection in the elderly population of Iran. The results of this meta-analysis indicate that the MMSE shows high diagnostic accuracy for dementia. However, it should be noted that there was significant heterogeneity among the included studies, which complicates the interpretation of the analysis results and the formulation of recommendations based on the pooled estimates. Additionally, there are essential factors to consider that may discourage the use of the MMSE in primary care settings. The MMSE and its modified version, the 3MS, are relatively lengthy assessments, which may limit their practicality in primary care settings where time constraints are common. Moreover, these tests may exhibit educational bias, disproportionately benefiting individuals with higher levels of education. Recognizing that these limitations could affect the equitable assessment and diagnosis of individuals with diverse educational backgrounds is crucial. Regarding the other cognitive tests evaluated in this study, namely, MoCA, ACE-III, PEACE, and RAVLT, their administration time is considerably long, rendering them impractical for routine use in primary care. Additionally, these tests may pose challenges in terms of interpretation, further limiting their suitability for primary care settings.

### Detection of cognitive impairment in specialized settings

Within specialized care settings, such as memory clinics or clinical trials, it is imperative to comprehensively evaluate various cognitive domains to identify even subtle impairments in cognitive function and evaluate treatment response. Therefore, there is a need for cognitive assessments that exhibit high diagnostic precision in detecting MCI and the early stages of dementia [70]. In the evaluation of cognitive screening instruments for older adults in Iran, the ACE-III emerged as the most accurate for diagnosing both MCI and dementia, with DORs of 147.57 and 1980, respectively. Absence of PPV and NPV data for ACE-III precluded NNSU calculation. Additionally, the MoCA showed DORs of 15.15 for MCI and 199.6 for dementia, with NNSU values indicating adequate performance for MCI (0.81) and excellent for dementia (0.56). Therefore, ACE-III or MoCA is recommended for older adults with over six years of education. For those with limited literacy, the PEACE test, with a DOR of 121.45, is recommended due to its significant diagnostic accuracy [65].

## Conclusion

This review examines the diagnostic accuracy of cognitive screening tools for detecting dementia and MCI in Iranian older adults across primary and specialized care settings. The findings suggest that the Qmci and RUDAS (for older adults with more than six years of formal education) and the AMTS, SPMSQ, and PMIS (for illiterate and low-educated individuals) are suitable screening tools in primary care due to their brevity, user-friendliness, and acceptable diagnostic accuracy.

In specialized care settings, ACE-III (for older individuals with a minimum of six years of formal education) and PEACE (for less educated older adults) are preferred screening tools due to their comprehensive assessment of cognitive domains and high diagnostic accuracy for dementia. However, it is important to acknowledge that these tools can be influenced by factors such as language, level of education, age, and physical abilities. Further research is needed to establish optimal cutoff scores considering these factors.

### Considerations and future directions in identifying the most accurate screening tool

The present study necessitates acknowledging certain considerations and outlining future directions for improving the identification of the most accurate cognitive screening tool. One concern arises regarding the potential overlap of subjects among the studies included in the analysis, particularly given that six of the studies sourced their samples from the memory clinic of Iran’s Dementia and Alzheimer’s Association (IDAA). This raises the possibility of overlapping primary study data, thereby introducing a potential bias. Furthermore, it is essential to acknowledge that the individuals served by the Alzheimer’s Association generally fall within the moderate and severe stages of the disease, which introduces the possibility of selection bias. Additionally, most of the studies employed a case‒control design without blinding, resulting in a significant bias in patient selection and flow and timing domains. The studies included in this analysis involved elderly participants with a wide range of literacy levels, from those with low literacy skills to those with higher education degrees. The participants’ ages also varied from 60 to over 90 years. These variations in literacy and age could potentially impact the participants’ cognitive performance and therefore affect the outcomes of the study. Finally, it is crucial to note that in clinical practice, relying solely on cognitive screening instruments to differentiate between normal cognition, MCI, and dementia without considering activities of daily living (ADL) functioning would yield restricted clinical utility.

Future research should incorporate nonduplicated subjects, broaden disease stages, and incorporate blinding techniques to reduce biases. Further exploration of the effects of literacy and age on cognitive performance is needed. Validating cognitive instruments across diverse populations is crucial for applicability and reliability. Large-scale studies to assess the precision of cognitive screening instruments in detecting mild cognitive impairment (MCI) in the Iranian population are recommended. Integration of daily living functioning assessments and comparative studies with identical populations can provide a comprehensive understanding of screening tests’ efficacy and practicality. Despite these considerations, this study provides valuable insights into the existing cognitive assessment tools and their suitability in various clinical and research settings.

### Electronic supplementary material

Below is the link to the electronic supplementary material.

Supplementary Material 1

## Data Availability

All data generated or analyzed during this study are included in this published article.

## Abbreviations

AMTS: Abbreviated Mental Test Score
ACE-III: Addenbrooke’s Cognitive Examination
AQT: A Quick Test of Cognitive Speed
CDT: Clock Drawing Test
DSM: Diagnostic and Statistical Manual of Mental Disorders
DOR: Diagnostic Odds Ratio
ICD: International Classification of Diseases
LR+: Positive Likelihood Ratio
LR-: Negative Likelihood Ratio
MCI: Mild Cognitive Impairment
MMSE: Mini–Mental State Examination
3MS: Modified Mini-Mental State Examination
MoCA: Montreal Cognitive Assessment
NPV: Negative Predictive Value) and PPV (Positive Predictive Value
OMIs: Outcome Measurement Instruments
PEACE: Persian Test of Elderly for Assessment of Cognition and Executive Function
PMIS: Picture-Based Memory Impairment Screen
PPV: Positive Predictive Value
NPV: Negative Predictive Value
Qmci: Quick Mild Cognitive Impairment Screen
QUADAS-2: Quality Assessment Tool for Diagnostic Accuracy Studies
RAVLT: Rey Auditory Verbal Learning Test
RUDAS: Rowland Universal Dementia Assessment
SPMSQ: Short Portable Mental Status Questionnaire
