# Supplementary material for: Diagnostic accuracy of cognitive screening tools validated for older adults in Iran: a systematic review and meta-analysis
Source: BMC Geriatr. 2024 May 14;24:428. doi: 10.1186/s12877-024-04963-w (PMC11095008; doi:10.1186/s12877-024-04963-w)
Supplement: Supplementary file 1 — Supplementary Material 1 [file 12877_2024_4963_MOESM1_ESM.docx]

**Appendix 1.**

Date of Search: March 2023

**Search Strategy for PubMed**

| **Results** | **Query** | **Number** |
| --- | --- | --- |
| **P (patients/participants/ population)** | | |
| 3,898 | **Search:** **("older adults" OR "elderly" OR "senior" OR "geriatric") AND ("Iran" OR "Iranian" OR "Farsi" OR "Persian")**  ("older adults"[All Fields] OR "elderly"[All Fields] OR "senior"[All Fields] OR "geriatric"[All Fields]) AND ("Iran"[All Fields] OR "Iranian"[All Fields] OR "Farsi"[All Fields] OR "Persian"[All Fields]) | #1 |
| **I (index tests/intervention)** | | |
| 8,604 | **Search: ("cognitive screening tools" OR "neuropsychological assessments" OR "cognitive tests")**  "cognitive screening tools"[All Fields] OR "neuropsychological assessments"[All Fields] OR "cognitive tests"[All Fields] | #2 |
| **C (comparator/reference tests)** | | |
| 257,014 | **Search: ("cognitive disorders" OR "cognitive impairment" OR "dementia" OR "MCI" OR "neurocognitive disorders")**  "cognitive disorders"[All Fields] OR "cognitive impairment"[All Fields] OR "dementia"[All Fields] OR "MCI"[All Fields] OR "neurocognitive disorders"[All Fields] | #3 |
| **O (outcome)** | | |
| 2,354,552 | **Search: ("diagnostic accuracy" OR "sensitivity" OR "specificity" OR "PPV" OR "NPV" OR "psychometric properties" OR "validation")**  "diagnostic accuracy"[All Fields] OR "sensitivity"[All Fields] OR "specificity"[All Fields] OR "PPV"[All Fields] OR "NPV"[All Fields] OR "psychometric properties"[All Fields] OR "validation"[All Fields] | #4 |
| 4 | **Search:** **(((("older adults" OR "elderly" OR "senior" OR "geriatric") AND ("Iran" OR "Iranian" OR "Farsi" OR "Persian")) AND (("cognitive screening tools" OR "neuropsychological assessments" OR "cognitive tests"))) AND (("cognitive disorders" OR "cognitive impairment" OR "dementia" OR "MCI" OR "neurocognitive disorders"))) AND (("diagnostic accuracy" OR "sensitivity" OR "specificity" OR "PPV" OR "NPV" OR "psychometric properties" OR "validation"))** | (#1 - #4) AND  Final Syntax |

**Search Filters:**

Document Type: Article

Time Span: Up to and including the year 2023.

Language: English and Persian

**Search Strategy for Web of Science**

| **Number** | **Query** | **Results** |
| --- | --- | --- |
| #1 | **Population (P)** | 20,606 |
|  | (TS=(aged OR elderly)) AND TS=(Iran OR Persian OR Farsi) |  |
| #2 | **Intervention/Index tests (I)** | 8,639 |
|  | TS=("cognitive screening tools" OR "neuropsychological assessments" OR "cognitive tests") |  |
| #3 | **Comparator/Reference tests (C)** | 297,278 |
|  | TS=("cognitive disorders" OR "cognitive impairment" OR "dementia" OR "MCI" OR "neurocognitive disorders") |  |
| #4 | **Outcome (O)** | 2,681,794 |
|  | TS=("diagnostic accuracy" OR "sensitivity" OR "specificity" OR "PPV" OR "NPV" OR "psychometric properties" OR "validation") |  |
| #5 | #1 AND #2 AND #3 AND #4 | 2 |

**Search Filters:**

Document Type: Article

Time Span: Up to and including the year 2023.

Language: English and Persian

**Search Strategy for SCOPUS**

| **Search Query** | **Results** |
| --- | --- |
| TITLE-ABS-KEY ( ( ( "older adults" OR "elderly" OR "senior citizens" OR "geriatric" ) AND ( "Iran" OR "Iranian" OR "Farsi" OR "Persian" ) AND ( "cognitive screening tools" OR "neuropsychological assessments" OR "cognitive tests" ) AND ( "cognitive disorders" OR "cognitive impairment" OR "dementia" OR "MCI" OR "neurocognitive disorders" ) AND ( "diagnostic accuracy" OR "sensitivity" OR "specificity" OR "PPV" OR "NPV" OR "psychometric properties" OR "validation" ) ) ) AND ( LIMIT-TO ( SRCTYPE , "j" ) ) AND ( LIMIT-TO ( SUBJAREA , "MEDI" ) OR LIMIT-TO ( SUBJAREA , "PSYC" ) OR LIMIT-TO ( SUBJAREA , "NEUR" ) ) AND ( LIMIT-TO ( DOCTYPE , "ar" ) ) AND ( LIMIT-TO ( LANGUAGE , "English" ) OR LIMIT-TO ( LANGUAGE , "Persian" ) ) AND ( LIMIT-TO ( EXACTKEYWORD , "Human" ) ) | 2 |

**Search Strategy for ScienceDirect**

| **Number** | **Search Query** | **Results** |
| --- | --- | --- |
| #1 | (elderly OR senior OR geriatric) AND (Farsi OR Iran OR Persian) | 26,667 |
| #2 | ("cognitive screening tools" OR "neuropsychological assessments" OR "cognitive tests") | 52,771 |
| #3 | ("cognitive disorders" OR "cognitive impairment" OR "dementia" OR "MCI" OR "neurocognitive disorders") | 517,756 |
| #4 | ("diagnostic accuracy" OR sensitivity OR specificity OR PPV OR NPV OR "psychometric properties" OR validation) | 1,000,000+ |
| #5 | #1 AND #2 AND #3 AND #4 | 19 |

**Search Filters:**

Document Type: Article

Time Span: Up to and including the year 2023.

**Search Strategy for the Scientific Information Database (SID), IranMedex, and the Iranian Research Institute for Information Science and Technology (IranDoc)**

| **Results** | **Search Query (Translated to Persian for optimal results)** |
| --- | --- |
| 11 | (elderly OR senior OR geriatric) AND (Farsi OR Iran OR Persian)AND ("cognitive screening tools" OR "neuropsychological assessments" OR "cognitive tests")AND ("cognitive disorders" OR "cognitive impairment" OR "dementia" OR "MCI" OR "neurocognitive disorders")AND ("diagnostic accuracy" OR sensitivity OR specificity OR PPV OR NPV OR "psychometric properties" OR validation) |
